# The role of Nurr1-miR-30e-5p-NLRP3 axis in inflammation-mediated neurodegeneration: insights from mouse models and patients’ studies in Parkinson’s disease

**DOI:** 10.1186/s12974-023-02956-x

**Published:** 2023-11-22

**Authors:** Tianbai Li, Xiang Tan, Lulu Tian, Congcong Jia, Cheng Cheng, Xi Chen, Min Wei, Yuanyuan Wang, Yiying Hu, Qiqi Jia, Yang Ni, Murad Al-Nusaif, Song Li, Weidong Le

**Affiliations:** 1 Liaoning Provincial Key Laboratory for Research On the Pathogenic Mechanisms of Neurological Diseases, The First Affiliated Hospital, Dalian Medical University, Dalian, 116021 China; 2 Institute of Neurology, Sichuan Academy of Medical Sciences, Sichuan Provincial Hospital, Chengdu, 610072 China

**Keywords:** Parkinson’s disease, Nuclear receptor related-1, Nucleotide-binding domain-like receptor protein 3, miRNA-30e-5p, Interleukin-1β

## Abstract

Nuclear receptor related-1 (Nurr1), a ligand-activated transcription factor, is considered a potential susceptibility gene for Parkinson’s disease (PD), and has been demonstrated to possess protective effects against inflammation-induced neuronal damage. Despite the evidence showing decreased *NURR1* level and increased pro-inflammatory cytokines in cell and animal models as well as in PD patients’ peripheral blood mononuclear cells (PBMCs), the underlying mechanism remains elusive. In this study, we investigated the molecular mechanism of Nurr1 in PD-related inflammation. Through the miRNA-sequencing and verification in PBMCs from a cohort of 450 individuals, we identified a significant change of a Nurr1-dependent miRNA miR-30e-5p in PD patients compared to healthy controls (HC). Additionally, PD patients exhibited an elevated plasma interleukin-1β (IL-1β) level and increased nucleotide-binding domain-like receptor protein 3 (NLRP3) expression in PBMCs compared to HC. Statistical analyses revealed significant correlations among *NURR1*, miR-30e-5p, and *NLRP3* levels in the PBMCs of PD patients. To further explore the involvement of Nurr1-miR-30e-5p-NLRP3 axis in the inflammation-mediated PD pathology, we developed a mouse model (Nurr1^flox+/Cd11b−cre+^, Nurr1^cKO^) conditionally knocking out Nurr1 in Cd11b-expressing cells. Our investigations in Nurr1^cKO^ mice unveiled significant dopaminergic neurodegeneration following lipopolysaccharide-induced inflammation. Remarkably, Nurr1 deficiency triggered microglial activation and activated NLRP3 inflammasome, resulting in increased IL-1β secretion. Coincidently, we found that miR-30e-5p level was significantly decreased in the PBMCs and primary microglia of Nurr1^cKO^ mice compared to the controls. Furthermore, our in vitro experiments demonstrated that miR-30e-5p specifically targeted NLRP3. In Nurr1-knockdown microglia, NLRP3 expression was upregulated via miR-30e-5p. In summary, our findings highlight the involvement of Nurr1-miR-30e-5p-NLRP3 axis in the inflammation-mediated neurodegeneration in PD, the results of which may offer promising prospects for developing PD biomarkers and targeted therapeutic interventions.

**Supplementary Information:**

The online version contains supplementary material available at 10.1186/s12974-023-02956-x.

## Introduction

Parkinson's disease (PD) is a progressive neurodegenerative disorder that is characterized by the loss of dopaminergic (DAergic) neurons in the substantia nigra pars compacta (SNc) and the presence of Lewy bodies, which are intracytoplasmic inclusions composed of α-synuclein aggregates [1]. PD is now viewed as a multi-system disorder with notable neuroinflammatory response mediated by microglia and synergizes with peripheral inflammation mediated by peripheral immune cells, thus promoting DAergic neurodegeneration [1, 2]. However, it is important to note that diverse phenotypes of the activated microglia can exert both degenerative and protective effects on neurons in a context-dependent manner [3, 4]. Therefore, a comprehensive understanding of the factors that influence microglial function and neuroinflammation in PD is essential for developing effective therapeutic strategies.

The ligand-activated transcription factor nuclear receptor related-1 (Nurr1, also known as NR4A2) is a potential susceptibility gene for PD, which is not only expressed in the central nerve system but also the central or the peripheral immune cells, such as microglia and peripheral blood mononuclear cells (PBMCs), respectively [5–7]. Nurr1 has a crucial role in the development and functional maintenance of DAergic neurons [8, 9]. Nurr1 has also been found to regulate neuroinflammation by inhibiting pro-inflammatory factors in microglia and astrocytes [10, 11]. Our recent in vivo study has demonstrated that selective deletion of Nurr1 in microglia potentiates lipopolysaccharide (LPS)-induced inflammatory insults to the DAergic neurons and results in age-dependent impairment of motor coordination and balance [12]. Additionally, case–control studies have shown that PD patients have significantly lower level of *NURR1* in PBMCs compared to age-matched healthy controls (HC) [13, 14]. Interestingly, *NURR1* expression in PBMC showed a negative correlation with the expression levels of inflammatory cytokines tumor necrosis factor-α (TNF-α), interleukin-1β (IL-1β), IL-4, IL-6, and IL-10 [14]. Therefore, the data from our studies and others strongly suggest that Nurr1 protects DAergic neurons from inflammation-induced degeneration in PD by regulating the inflammatory response. Gaining further insight into the Nurr1 signaling cascade underlying its anti-inflammatory effect holds great importance for the development of valuable strategies for PD diagnosis and therapy.

Within the realm of neuroinflammation pathways identified in PD, significant attention has been directed toward the activation of the nucleotide-binding domain-like receptor protein 3 (NLRP3) inflammasome. NLRP3 is primarily situated within inflammatory cells, and its activation is associated with the induction of a sterile inflammatory response, contributing to the pathological processes in PD [15]. The activation process involves priming with NLRP3 protein induction as its central component, followed by the oligomerization of apoptosis-associated speck-like protein (ASC) and the subsequent activation of pro-Caspase-1 [15]. The active Caspase-1 cleaves pro-IL-1β into its cleaved form, IL-1β, which is subsequently secreted from microglia [16]. Despite the growing interest in therapeutically targeting the NLRP3 inflammasome for PD treatment, a knowledge gap persists concerning the effector mechanisms that instigate NLRP3 transcription. microRNAs (miRNAs), as pivotal modulators of gene expression, wield post-transcriptional control over genes implicated in PD pathogenesis [17–19]. However, the intricacies of the regulatory network involving miRNAs in inflammation remain incompletely elucidated. Transcription factors have emerged as potential orchestrators of target miRNA expression by binding to miRNA gene DNA, offering a novel perspective for investigating the upstream regulation of miRNAs [20]. Consequently, we postulate that Nurr1 may wield regulatory control over NLRP3 expression, thereby influencing the activation of the NLRP3 inflammasome within the inflammatory context of PD, via a transcription factor-miRNA regulatory pathway.

In the current study, we first identified miR-30e-5p as a Nurr1-dependent miRNA in the inflammatory process of PD using miRNA-sequencing. Then, we found significant changes in miR-30e-5p and *NLRP3* expression levels in a relatively large sample size of PBMC from PD patients (n = 224) compared to HC (n = 226), showing a correlation with the expression level of Nurr1. Subsequently, we generated a Nurr1 conditional knockout mouse model (Nurr1^flox+/Cd11b−cre+^, Nurr1^cKO^) with Nurr1 deficiency in Cd11b-expressing cells, including PBMC and microglia. Our investigation in Nurr1^cKO^ mice unveiled that the absence of Nurr1 in PBMC and microglia led to significant PD-related pathology and inflammation via Nurr1-miR-30e-5p-NLRP3 axis. Furthermore, we provided compelling in vitro evidence of the molecular mechanism underlying the Nurr1-miR-30e-5p-NLRP3 axis in microglia. Our findings from the mouse model and patient studies highlight the involvement of the Nurr1-miR-30e-5p-NLRP3 axis in inflammation-mediated neurodegeneration in PD.

## Materials and methods

### Participants, blood sampling, plasma and PBMCs separation

A total of 450 participants, including 224 PD patients and 226 HC individuals, were enrolled between January 2018 and June 2021 (Table 1). The PD patients in this study were recruited from individuals visiting the Neurology Department of the First Affiliated Hospital of Dalian Medical University. The diagnosis of idiopathic PD in these patients was based on the Movement Disorder Society Clinical Diagnostic Criteria for PD [21]. Among the 224 PD patients, 107 had recent-onset PD without receiving any PD treatment, while the remaining 117 patients were under PD medications. The severity of PD was assessed using the Modified Hoehn and Yahr (H-Y) staging [22]. PD patients with other major neurological disorders, ongoing infectious/autoimmune conditions, or serious metabolic disorders were excluded from the study. HC participants were recruited from the Health Examination Center at the First Affiliated Hospital of Dalian Medical University, and they showed no evident neurological or non-neurological disorders. The study received ethical approval from the Ethics Committee of the First Affiliated Hospital of Dalian Medical University (approval number: LCKY2014-29), and written informed consent was obtained from all participants.

**Table 1:** Demographics and clinical characteristics of the participants in this study

Characteristics	NLRP3&miRNA study cohort (n = 450)		*p* value	IL-1β study cohort (n = 228)		*p* value	miRNA sequencing cohort (n = 10)		*p* value	Correlation analysis cohort	*p* value
	HC (n = 226)	PD (n = 224)		HC (n = 112)	PD (n = 116)		HC (n = 5)	PD (n = 5)		PD (n = 98)	
Age ^a^	67.39 ± 9.71	67.35 ± 9.84	NS^b^	67.47 ± 9.08	67.5 ± 9.58	NS^b^	72 ± 4.6	72.2 ± 4.32	NS^b^	68.33 ± 9.64	NS^b^
Gender (M: F)	135: 91	133: 91	NS^c^	66: 46	69: 47	NS^c^	3: 2	3: 2	NS^c^	58: 40	NS^c^
Disease duration (years)^a^	NA	5.76 ± 4.2	NA	NA	5.8 ± 4.3	NA	NA	7.8 ± 7.56	NA	5.69 ± 4.06	NA
Hoehn and Yahr score^a^	NA	2.3 ± 1.03	NA	NA	2.26 ± 0.74	NA	NA	2.5 ± 1.1	NA	2.28 ± 0.8	NA

*PD* Parkinson’s disease, *HC* healthy control, *NS* not significant, *NA* not analyzed

^a^Data are expressed as mean ± SD

^b^Indicates generated by the Kruskal–Wallis test

^c^Indicates generated by the chi-square test

Peripheral blood samples were collected through direct venipuncture, drawing 2 ml of peripheral blood from the cubital vein into blood collection tubes containing ethylene diamine tetra-acetic acid. Subsequently, plasma was separated by aliquoting 800 µl into sterile tubes and stored at − 80 °C. PBMCs were isolated from the Peripheral Lymphocyte Separation Medium (HAOYANG, China) by centrifugation at 500×*g* for 20 min and stored at − 80 °C.

### The generation of conditional knockout Nurr1 mouse model

The floxed Nurr1 knock-in mice (Nurr1^flox/flox^) were bred with Cd11b-Cre driver mice [Tg(ITGAM-cre)2781Gkl] to generate homozygous floxed, Cre-positive, Nurr1 mice (Nurr1^cKO^), as previously described [12]. Heterozygous mice (Nurr1^flox/wt^) with a C57BL/6J background were generated by Shanghai Model Organisms Center, Inc. (Shanghai, China). Control mice referred to littermates of homozygous floxed, Cre-negative, Nurr1 mice (Nurr1^flox+/cd11bcre−^, Nurr1^cWT^). All mice were housed under standard conditions with a room temperature of 22 ± 2 °C air exchange, and a 12-h light/dark cycle, with ad libitum access to food and water. All animal care and procedures were conducted in accordance with the Laboratory Animal Care Guidelines approved by the Institutional Animal Care Committee at Dalian Medical University.

For genotyping, Nurr1^flox/flox^ mice were identified using PCR assay (2XEasyTaq PCR SuperMix, Transgen Biotech, China) on tail biopsy samples. The wild-type allele exhibited an amplification product size of 662 bp, while the homozygous flox allele (Nurr1^flox/flox^) displayed a length of 766 bp. Cd11b-cre transgenic mice were identified by the presence of a 445 bp target amplicon (Additional file 1: Figure S1A). The genotyping primers were summarized in Additional file 1: Table S2.

### Primary microglia isolation and culture

Primary microglia were isolated from the cortex of postnatal days 4–5 Nurr1^cKO^ pups and their respective littermate controls. Following meningeal removal, brain tissue was minced and subjected to trypsin and DNase I digestion (Solarbio, Beijing, China). The resulting cell mixture in F12 media was passed through a 70 μm cell strainer into conical tubes. Subsequently, cells were resuspended in F12 media supplemented with 10% fetal calf serum, and then seeded into 75 cm^2^ flasks. Upon reaching confluence (10–14 days), the flasks were gently shaken at 120 rpm for 2 h at 37 °C, facilitating the collection of floating cells, which were then allowed to adhere to the flask overnight. Finally, the adherent cells were harvested and employed for subsequent experimental procedures.

### LPS administration paradigms

Nurr1^cKO^ mice and their age-matched Nurr1^cWT^ littermates at nine months of age were intraperitoneally injected with either PBS or 0.33 mg/kg LPS (Escherichia coli, Sigma) at the designated time of day, twice a week, for a duration of 1 month (n = 6 per group) [12]. Mouse body weights were measured twice a week. Following a 2-month resting period, the mice were euthanized in a humane manner. The brains were isolated and subsequently processed for biochemical or histological studies.

### Cell culture and transfection

The immortalized RAW264.7 macrophage cells, microglial BV2 cells and HEK 293T cells used in this study were obtained from ATCC and cultured in DMEM (Gibco, Gaithersburg, MD, USA) supplemented with 10% FBS (Gibco), Penicillin (100 U/ml)-streptomycin (100 mg/ml) (Sigma–Aldrich, St. Louis, MO, USA), and maintained at 5% CO_2_ and 37 °C. Cell detachment was achieved by treating the cells with 0.25% Trypsin EDTA (ThermoFisher Scientific, Waltham, MA) solution. To establish BV2 cells stably expressing Nurr1-shRNA, BV2 cells were transfected with HBLV-ZsGreen-PURO and HBLV-h-NR4A2-shRNA1-ZsGreen-PURO (Hanbio, Shanghai, China) using lipofectamine 6000 (Beyotime, Shanghai, China) according to the manufacturer’s instructions. Forty-eight hours post-transfection, the cells were subjected to selection using a cell culture medium containing 2 μg/ml of puromycin (Sigma–Aldrich, St. Louis, MO, USA). Real-time quantitative PCR (RT-qPCR) and Western blotting confirmed the successful knockdown of Nurr1. miR-30e-5p mimics/inhibitors and miRNA controls were purchased from Ruibio (Ruibio, China). The pmirGLO Dual-Luciferase miRNA Target Expression system, which contains the wild-type sequence of NLRP3’s 3′-untranslated region (UTR) or mutant sequence, as well as the pGL3 Luciferase Reporter system (pre-miR-30e-WT and pre-miR-30e-Mut plasmids), were constructed and synthesized by Miaoling (Miaoling, China). Additionally, Nurr1 overexpressing plasmid [pcDNA3.1(−)-Nurr1, Nurr1 OE] and control plasmid (pcDNA-3.1, OE-NC) were generated as previously described [23]. For all experiments, frozen stocks of cells were revived and cultured for three generations before being used.

### miRNA sequencing (miRNA-seq)

miRNA-seq was performed by Aksomics (Shanghai, China) [24]. Briefly, a library was generated from the identified RNAs, and its quality was assessed using an Agilent 2100 Bioanalyzer. miRNA-seq was then conducted using an Illumina NextSeq 500 sequencing platform (Illumina). Following screening the original reads with Solexa CHASTITY to obtain clean reads, known miRNAs were quantified, and new miRNAs were predicted using miRDEEP2. Differential expression analysis of miRNAs was carried out using R software.

### Total RNA extraction and RT-qPCR quantification

RNA was extracted from cells and mouse brain tissue using mirVana mRNA Isolation Kit (Ambion, Carlsbad, CA, United States) and RNA Easy Fast Tissue/Cell Kit (TIANGEN, Beijing, China) according to the manufacturer’s instructions. For TaqMan RT-qPCR cDNA was produced using a target-specific probe, the TaqMan microRNA reverse transcription kit (Applied Biosystems, CA, United States) and the Bio-Rad iCycler Thermal cycler. The relative expression levels of miRNAs were measured using TaqMan™ Universal mix, with specific primers for miR-21-5p, miR-30e-5p, miR-29a-5p, miR-105-5p and the reference gene U6 obtained from Applied Biosystems. The other target gene’s mRNA levels were then measured by quantitative real-time PCR (qRT-PCR). The PCR reactions were performed as described in detail previously [14]. The specific primers targeting PBMCs are presented in Additional file 1: Table S2. The data regarding the expression level of *NURR1* mRNA in PBMC of PD patients and HC were obtained from our previous study [14]. The target gene expression was determined using the 2^−delta Ct^ method.

### Cytokine measurement

A total of 228 human plasma samples were randomly selected from the participants in this study, with 112 samples obtained from HC and 116 samples obtained from patients with PD. The plasma samples were simultaneously separated during the extraction of PBMCs. The concentration of cleaved IL-1β in the plasma was measured using the BD OptEIA™ Human IL-1β ELISA KIT II (BD Biosciences, San Diego, CA, USA), following the manufacturer's instructions. Similarly, the production of IL-1β in the midbrain of Nurr1^cKO^ mice and control mice was assessed using the Mouse IL-1β ValukineTM ELISA Kit (VAL601, Bio-Techne, China), as per the manufacturer’s instructions. The concentrations of IL-1β were determined by comparing the optical density (OD) values of the samples to the standard curves provided in the respective ELISA kits.

### Western blotting

Brain tissue from mice and cultured cells were resuspended in RIPA lysis buffer (Beyotime Biotechnology, Shanghai, China) containing protease inhibitor cocktails (Sigma-Aldrich, St. Louis, MO, USA) and then lysed on ice for 30 min and centrifuged at 12,000×*g* for 15 min. The protein concentration in the supernatant was detected by a BCA Protein Assay kit (Beyotime Biotechnology, Shanghai, China). 40 μg of total protein were subjected to SDS–polyacrylamide gel electrophoresis and then transferred to 0.45 μm polyvinylidene difluoride membranes (Millipore, Burlington, MA, USA). After blocking with 5% skimmed milk for one hour at room temperature, membranes were incubated with appropriate primary antibodies at 4 °C overnight, followed by a 1-h incubation with a peroxidase-conjugated secondary antibody. The primary antibodies used are detailed in Additional file 1: Table S1. The protein bands were then detected with the enhanced chemiluminescence detection kit (Wanlei Biotechnology, Shenyang, China) and quantified using the FluorChem Q system (ProteinSimple, California, USA).

### High-performance liquid chromatography (HPLC)

Mouse brains were rapidly removed, and the right striatum was isolated using a tissue punch. The stratum specimen was weighed and sonicated on ice for HPLC, which was performed as described in detail previously [12]. Different DA standard concentrations were detected to plot a standard curve for data analysis.

### Immunofluorescent staining

Mouse brains were collected at indicated time points. The brains were rapidly isolated and postfixed in ice-cold 4% paraformaldehyde and subsequently dehydrated for 24 h in 15% and 30% sucrose at 4 °C, as described previously. Sections were immersed in a blocking solution (10% normal goat serum, 0.25% Triton-X 100, and 0.05% NaN3 in PBS) overnight at 4 °C. Subsequently, they were incubated with the primary antibodies overnight at 4 °C, with the list of primary antibodies available in Additional file 1: Table S1. Finally, the stained sections were visualized and photographed with a confocal microscope [A1 confocal, Nikon Instruments (Shanghai) Co., Ltd.]. The paired images in the figures were collected at the same gain and offset settings.

### Image analysis

For counting DAergic neurons, a set of coronal sections (40 μm per section, with a spacing of three sections from Bregma − 2.70 to − 3.88 mm) were subjected to anti-TH staining for quantification. Quantitative data were obtained from 8 to 10 sections per animal. Immunofluorescent staining (IFC) was analyzed using ImageJ2 software (version 2.9) to quantify the number of puncta, with data collected from 3 to 4 slices per animal.

### Dual-luciferase reporter assays

The wild-type sequence of NLRP3’s 3′UTR (3′UTR-NLRP3-WT) or mutant sequence (3′UTR-NLRP3-Mut) within the binding sites of the pre-miR-30e-5p were cloned into the pmirGLO Dual-Luciferase miRNA Target Expression Vector (Promega). Firefly luciferase served as the primary reporter to assess miRNA regulation of the 3′UTR, while Renilla luciferase acted as an internal control for normalization. HEK 293T cells were seeded in a 24-well plate and co-transfected with luciferase reporters and 20 nM of either miR-30e mimics or miR-NC. Furthermore, to investigate the interaction of Nurr1 and the pre-miR-30e promotors, the wild type or mutated binding sites of the pre-miR-30e-5p promoter, contained with the pGL3 Luciferase Reporter Plasmid (pre-miR-30e-WT/pre-miR-30e-Mut), were co-transfected with the control pRL-TK plasmid into 293T cells along with the Nurr1-OE plasmid. Luciferase activities were measured 48 h post-transfection using the Dual-Glo Luciferase Assay System (Beyotime, China), and the relative luciferase activity was quantified and normalized to Renilla activity.

### Chromatin immunoprecipitation-qPCR

Chromatin immunoprecipitation (ChIP) was performed using a commercial ChIP assay kit (Beyotime, China) according to the manufacturer’s protocol. Briefly, BV2 cells were crosslinked with 1% formaldehyde, sonicated with PBS containing 1 mM phenylmethylsulfonyl fluoride, and centrifuged at 12,000×*g* for 5 min. Equal amounts of total proteins were added 1 μl of normal rabbit anti-IgG (Millipore, Burlington, MA, USA) and 10 μg of anti-Nurr1 (Abcam, Cambridge, MA, USA) antibodies. Following immunoprecipitation, chromosomal DNA was purified using a DNA purification kit (Beyotime, China). The promoter regions of pre-miR-30e-5p were detected using RT-qPCR, with the primer details provided in Additional file 1: Table S2.

### Statistical analysis

Quantitative data were expressed as mean ± SEM or mean ± SD for each group. Two-way analysis of variance (ANOVA) followed by Tukey’s post-test was used for analyses across multiple groups. We used the unpaired Student T test for experimental designs with less than three groups and one variable. Spearman’s coefficients were calculated to evaluate bivariate associations between expression levels of *NURR1*, miR-30e-5p, *NLRP3* and IL-1β. Partial correlation analyses were utilized to adjust for age, sex, and pharmacotherapy. Receiver operating characteristic (ROC) curves and areas under the curves (AUC) were used to evaluate the diagnostic performance of the potential biomarkers and were carried out with the SPSS software version 26.0 (SPSS Inc., Chicago, IL, United States). Other statistical analyses were performed with Prism software (GraphPad software version 9.0). No statistical methods were used to predetermine sample size, but our sample sizes are similar to those reported in previous publications. A *p*-value < 0.05 was considered as statistical significance.

## Results

### miRNA expression profiling identifies Nurr1-dependent signature miRNA in the inflammation pathway of PD

**Fig. 1:**
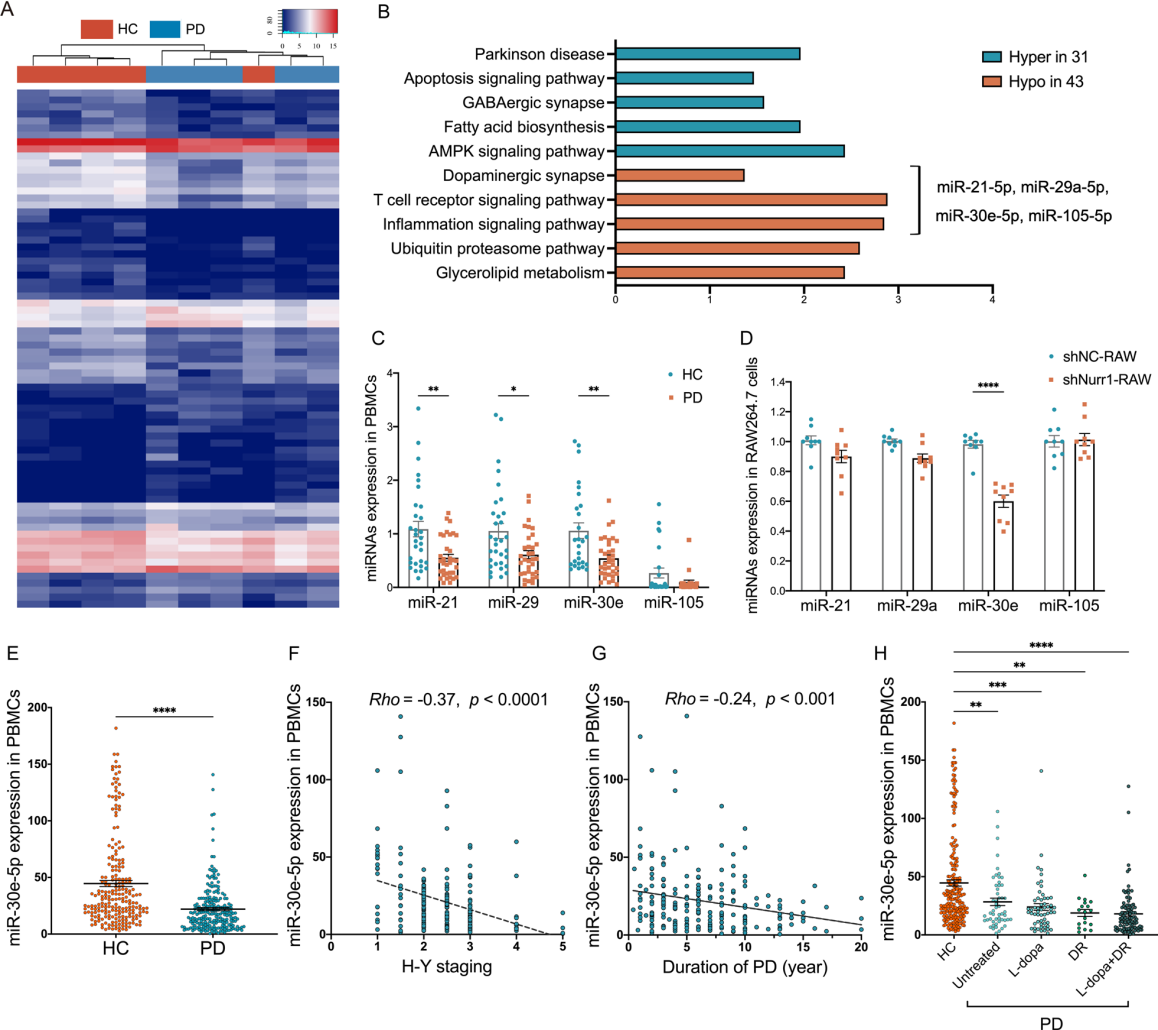
miRNA expression profiling identified Nurr1-dependent signature miRNA in the inflammatory process of PD. **A** Expression heatmap of differential miRNA profiles in the PBMCs of HC (n = 5) and PD patients (n = 5) by miRNA sequencing, indicating significant disease-based clustering. **B** The representative KEEG terms of differentially expressed miRNAs in PD patients. **C** qRT-PCR was used to detect the expression levels of miR-21-5p, miR-30e-5p, miR-29a-5p and miR-105-5p in the PBMCs of PD patients (n = 42) and HC (n = 44). Data are presented as mean ± SEM. *p < 0.05, **p < 0.01. Unpaired Student T test. **D** qRT-PCR analysis of miR-21-5p, miR-30e-5p, miR-29a-5p and miR-105-5p in the stable Nurr1-knockdown RAW264.7 macrophage cells (ShNurr1-RAW) compared with the control RAW264.7 cells (ShNC-RAW). ****p < 0.0001. Unpaired Student T test. **E** Scatter plots of miR-30e-5p expression level in the PBMCs of HC (n = 226) and PD (n = 224). The effects of **F** H-Y staging, **G** duration of disease and **H** medication on the expression level of miR-30e-5p in PBMCs of PD patients. Horizontal bars represent mean and SEM values. **p < 0.01, ***p < 0.001, and ****p < 0.0001 by Unpaired Student T test. *Rho*, correlation coefficient by Spearman correlation analysis. *DR* DA receptor agonists monotherapy, *l-dopa* *l*-dopa monotherapy, *l-dopa + DR* the combination of DA agonists and l-dopa

To identify miRNAs potentially involved in the inflammatory process of PD, we conducted a screening of miRNAs associated with neuroinflammation in PD. By performing miRNA-seq on PBMCs samples from PD patients (n = 5) and HC (n = 5, Table 1), we discovered 74 significantly differentially expressed miRNAs in PD patients. Among them, 31 miRNAs were up-regulated and 43 miRNAs were down-regulated (Fig. 1A). Further analysis of miRNA Kyoto Encyclopedia of Genes and Genomes (KEEG) pathway [25] revealed a significant enrichment of miRNAs involved in multiple physiology and pathophysiology processes in PD, including inflammation, T cell receptor signaling, ubiquitin–proteasome, AMPK signaling, glycerolipid metabolism, fatty acid biosynthesis, γ-aminobutyric acid (GABA)-ergic synapse, DAergic synapse, and apoptosis signaling pathways (Fig. 1B). Additionally, we identified miR-21-5p, miR-30e-5p, miR-29a-5p, and miR-105-5p as significantly dysregulated miRNAs overlapping in inflammation, T cell receptor signaling and DAergic synapse pathways in PD (Fig. 1B). To validate our findings, we performed RT-qPCR and confirmed that expression levels of miR-21-5p, miR-30e-5p, miR-29a-5p, and miR-105-5p were significantly reduced in the PBMCs of PD patients (n = 46) compared to HC (n = 43, *p* < 0.05) (Fig. 1C).

To identify Nurr1-dependent signature miRNAs among these miRNAs, we evaluated their expression levels in the Nurr1-knockdown RAW264.7 macrophage cells (ShNurr1-RAW) and control cells (ShNC-RAW) using RT-qPCR. The results showed a significant decrease in miR-30e-5p expression in ShNurr1-RAW264.7 cells compared to ShNC-RAW264.7 (*p* < 0.0001), while miR-21-5p, miR-29a-5p, and miR-105-5p showed no change (Fig. 1D).

Subsequently, we attempted to validate the substantial changes in miR-30e-5p expression between PD patients and HC, utilizing a relatively large sample size (n = 450). The demographics and disease characteristics of the PD patients and HC are outlined in Table 1. RT-qPCR analyses revealed a significant alteration in the expression level of miR-30e-5p in the PBMCs of PD patients (n = 224) compared to HC (n = 226, *p* < 0.0001) (Fig. 1E). Correlation analyses revealed significant negative associations between miR-30e-5p and H-Y score (*Rho* = -0.37, *p* < 0.0001, Fig. 1F), as well as between miR-30e-5p and disease duration (*Rho* = -0.24, *p* < 0.001, Fig. 1G), implying that the reduced expression of miR-30e-5p may contribute to the progression of PD and serves as a marker for evaluating its severity. These findings collectively underscore the significant pathological involvement of miR-30e-5p in PD and suggest its potential role as a Nurr1-dependent signature miRNA implicated in the neuroinflammatory processes associated with PD.

### NLRP3 inflammasome is activated in the peripheral blood of PD patients

**Fig. 2:**
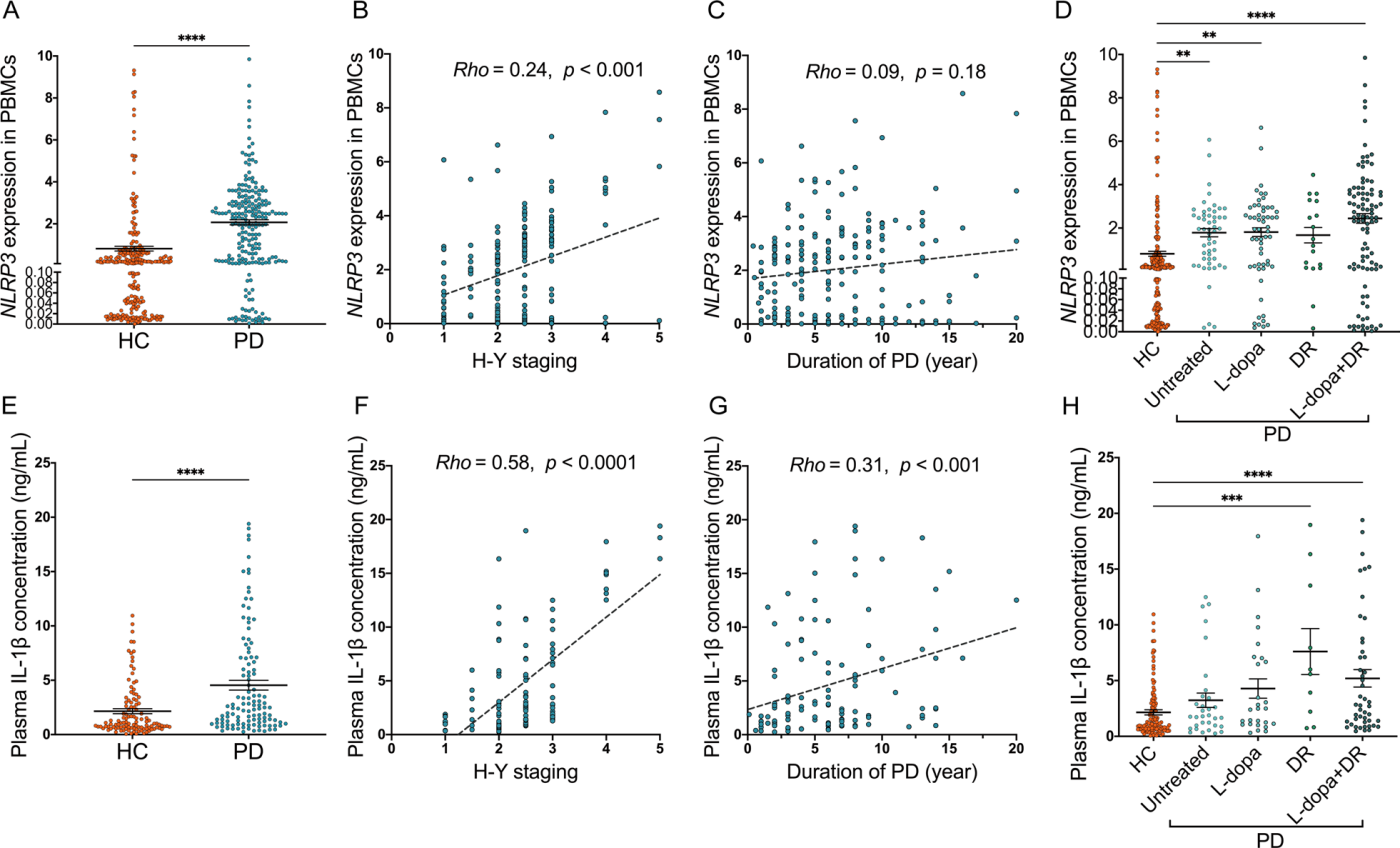
NLRP3 inflammasome is activated in the peripheral blood of PD patients. **A** Scatter plots of *NLRP3* relative mRNA expression level in the PBMCs of HC (n = 226) and PD (n = 224). The effects of **B** disease severity (H-Y staging), **C** disease course (duration of PD) and **D** medication on the expression level of *NLRP3* in PBMCs of PD patients. **E** Scatter plots of the cleaved form of IL-1β concentration in the plasma of HC (n = 112) and PD (n = 116). The effects of **F** H-Y staging, **G** duration of disease and **H** medication on IL-1β concentration in the plasma of PD patients. Horizontal bars represent mean and SEM values. **p < 0.01, ***p < 0.01, and ****p < 0.0001 by unpaired Student T test. *Rho*, correlation coefficient by Spearman correlation analysis. *DR* DA receptor agonists monotherapy, *l-dopa* l-dopa monotherapy, *l-dopa + DR* the combination of DA agonists and l-dopa

Owing to the elevated expression levels of pro-inflammatory cytokines in the PBMCs of PD patients compared to HC [14], and considering that NLRP3 has been proposed as a target gene of miR-30e-5p [26], our subsequent objective was to determine whether the NLRP3 inflammasome is activated in PD patients. We collected a total of 450 human peripheral blood samples, consisting of 450 PBMCs (224 from patients with idiopathic PD and 226 from HC) and 228 plasma samples (116 from patients with idiopathic PD and 112 from HC). The demographics and disease characteristics of the cohort were summarized in Table 1. No significant differences in sex or age were observed between the PD and HC. We conducted qRT-PCR to evaluate the *NLRP3* mRNA level in the PBMCs of PD patients and HC. Our results showed a significantly higher *NLRP3* in the PBMC of PD patients than that of HC (*p* < 0.0001, Fig. 2A). Spearman’s correlation analyses revealed a significant correlation between disease severity (as measured by the H-Y score) of PD and the expression level of *NLRP3* (*Rho* = 0.24, *p* < 0.001, Fig. 2B). However, no significant association was found between disease duration (years after the onset of disease symptoms) and the *NLRP3* gene level (*Rho* = 0.09, *p* = 0.18, Fig. 2C). Additionally, we assessed the concentrations of cleaved IL-1β in the plasma of PD and HC groups. Results obtained from ELISA indicated that the plasma IL-1β concentration in PD patients was significantly higher than that in HC (*p* < 0.0001, Fig. 2E). Spearman’s coefficients between the H-Y score and IL-1β concentration in the PD were 0.58 (*p* < 0.0001, Fig. 2F). Moreover, disease duration exhibited a significant correlation with IL-1β concentration (*Rho* = 0.31, *p* < 0.001, Fig. 2G). Our data further demonstrated that different PD pharmacotherapy regimens had no discernible effect on the expression of *NLRP3* and IL-1β (Fig. 2D, H). In summary, these results indicate that the NLRP3 inflammasome is activated in the peripheral blood of PD patients and is correlated with the motor severity of PD.

### Associations between the expression levels of NURR1, miR-30e-5p, NLRP3 and IL-1β in PD patients

**Fig. 3:**
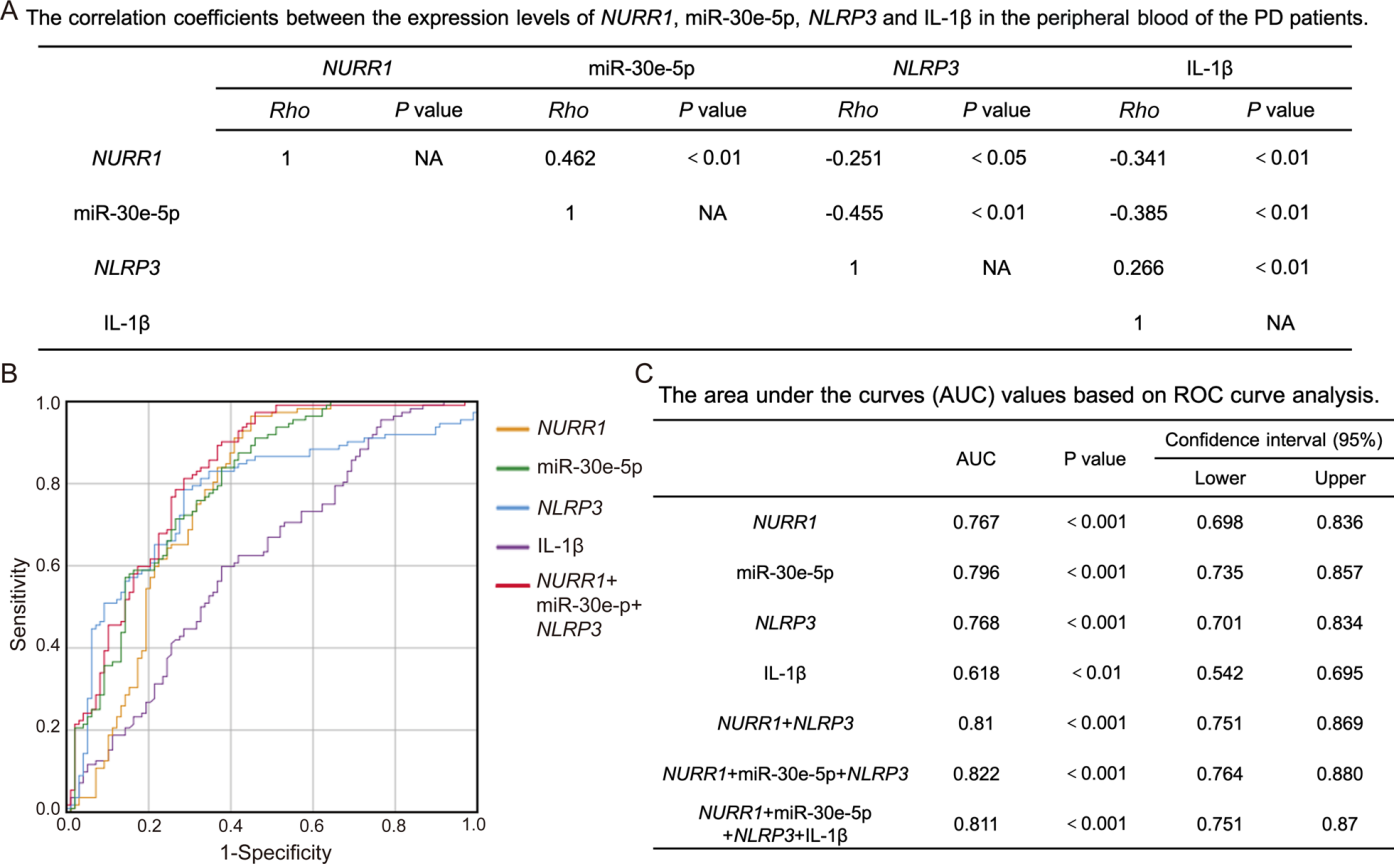
Associations between the expression levels of *NURR1*, miR-30e-5p, *NLRP3* and IL-1β in PD patients. **A** Table of correlation coefficients between PBMC *NURR1*, miR-30e-5p and *NLRP3* expression levels and plasma IL-1β concentration of PD patients using Spearman’s correlation analysis. *Rho*, correlation coefficient. **B** Receiver operating characteristic (ROC) curves of *NURR1*, miR-30e-5p, *NLRP3* and IL-1β levels for PD versus HC. **C** Table of the area under curves (AUC) values in *NURR1*, miR-30e-5p, *NLRP3*, IL-1β and their combinations using ROC curve analysis

To further validate the relationships among the expression levels of *NURR1*, miR-30e-5p, *NLRP3* and IL-1β in PD patients, we conducted an analysis using Spearman's coefficients to examine the correlations between PBMC *NURR1*, miR-30e-5p and *NLRP3* expression levels, as well as plasma IL-1β level. The demographic characteristics of the PD patient cohort, who were assessed for the levels of *NURR1*, miR-30e-5p, *NLRP3*, and IL-1β, were summarized in Table 1. The correlation coefficients demonstrated significant associations among levels of *NURR1*, miR-30e-5p, *NLRP3*, and IL-1β in PD patients (Fig. 3A). Specifically, the expression level of *NURR1* exhibited a positive association with the expression level of miR-30e-5p (*Rho* = 0.462, *p* < 0.01) and an inverse relationship with the levels of *NLRP3* (*Rho* = − 0.251, *p* < 0.05) and IL-1β (*Rho* = − 0.341, *p* < 0.01, Fig. 3A). Furthermore, the level of miR-30e-5p in PBMCs showed an inverse correlation with both the *NLRP3* level (*Rho* = − 0.455, *p* < 0.01) and plasma IL-1β level (*Rho* = − 0.385, *p* < 0.01) in PD (Fig. 3A). Additionally, the expression level of *NLRP3* exhibited a significant correlation with IL-1β in PD (*Rho* = 0.266, *p* < 0.01, Fig. 3A).

The diagnostic performance of PBMCs *NURR1*, miR-30e-5p, *NLRP3*, and plasma IL-1β for PD was evaluated using the area under the curve (AUC) values based on the analysis of the receiver operating characteristic (ROC) curve (Fig. 3B, C). The results showed that the combination of *NURR1*, miR-30e-5p, and *NLRP3* exhibited superior AUCs for PD, with a value of 0.82 (95% CI, 0.764–0.88, *p* < 0.01), compared to individual biomarkers (*NURR1*, miR-30e-5p, *NLRP3*, and IL-1β) or other combinations (Fig. 3B, C). These findings may predict a NURR1-miR-30e-5p- NLRP3 axis in the inflammation-related DAergic neurodegeneration in PD. Our data further suggest that the combination of *NURR1*, miR-30e-5p, and *NLRP3* may have a diagnostic value for PD.

### Nurr1 conditional knockout mouse model exhibits DAergic neurodegeneration

To further confirm the involvement of NURR1-miR-30e-5p-NLRP3 axis in PD in vivo, we generated a mouse model (Nurr1^cKO^) with specific Nurr1 deficiency in Cd11b-expressing cells. Cd11b is an integrin expressed exclusively in the myeloid lineages, including monocytes and macrophages, and widely used for genetic targeting of central immune cells, microglia. The Cre-Loxp-mediated recombination system, driven by the Cd11b promoter, led to the knockout of the exon 3 of Nurr1 genomic DNA. Nurr1^cKO^ mice were identified through tail genotyping (Additional file 1: Figure S1A), and exhibited an 87% reduction in Nurr1 mRNA level in the PBMCs (Additional file 1: Figure S1B) and a 91% reduction of Nurr1 in isolated primary microglia from the cortex of newborn pups (Additional file 1: Figure S1B). Moreover, IFC results demonstrated negligible expression of Nurr1 in Iba-1-positive microglia in the SNc of 2-month-old Nurr1^cKO^ mice (Additional file 1: Figure S1C). This evidence indicates the successful knockout of Nurr1 in the PBMCs and microglia within the Nurr1^cKO^ mouse model.

**Fig. 4:**
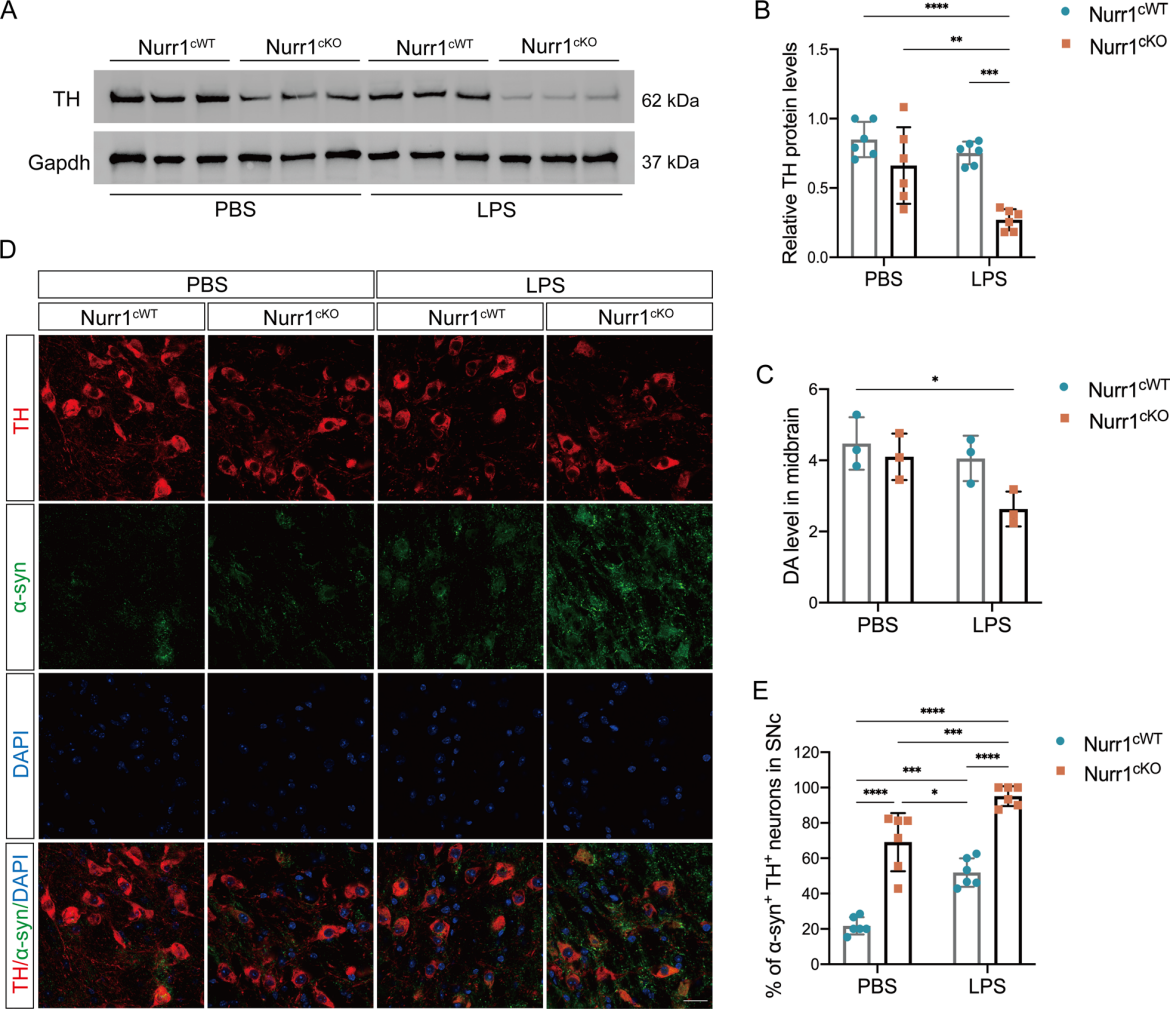
DAnergic neurodegeneration in Nurr1^cko^ mice. **A** Representative immunoblots and quantitative analysis **B** of TH level in the midbrain of 12-month-old Nurr1^cKO^ and Nurr1^cWT^ mice (n = 3 mice per each group) after LPS or PBS treatment. **C** HPLC analysis of DA level in the midbrain of mice after LPS or PBS injection (n = 3 mice per each group). **D** Double-label immunostaining of α-synuclein (green) and TH (red) in the SNc of the 12-month-old mice after PBS or LPS treatment. Scale bar = 20 μm. **E** The proportion of α-synuclein positive and TH-positive neurons (α-synuclein^+^-TH^+^) in the SNc was quantified (n = 3 mice per group). *p < 0.05, **p < 0.01, ***p < 0.001, ****p < 0.0001, Two-way ANOVA Tukey test

We proceeded to analyze the impact of Nurr1 deficiency on the neuron-microglia cross-talk. Our previous research revealed that deleting Nurr1 in microglia does not result in a significant loss of DAergic neurons in 12-month-old Nurr1^cKO^ mice. However, exposure to LPS can significantly amplify the loss of DAergic neurons in this Nurr1^cKO^ mouse model [12]. Consequently, we administered intraperitoneal injections of LPS to Nurr1^cKO^ and Nurr1^cWT^ mice as previously described [12]. To quantify the DAergic neurons, we conducted Western blotting analysis of tyrosine hydroxylase (TH), a representative marker for DAergic neurons. The results showed a decrease in TH protein level in the midbrain of 12-month-old Nurr1^cKO^ mice after LPS treatment, compared to age-matched Nurr1^cWT^ mice treated with LPS or Nurr1^cKO^ mice treated with PBS (*p* < 0.01, Fig. 4A, B). Furthermore, HPLC analysis showed a reduction in DA concentration within the striatum of Nurr1^cKO^ mice following LPS administration compared to the control mice (*p* < 0.05, Fig. 4C). These results suggest that the absence of Nurr1 in microglia intensifies LPS-induced inflammatory damage to the nigrostriatal DAergic neurons.

To further investigate the effect of Nurr1 deficiency in microglia on PD-related pathology, we examined the expression of endogenous α-synuclein by IFC. We conducted double immunostaining of pS129-α-synuclein (pS129αSYN) and TH, revealing an increased co-localization of α-synuclein in the DAergic neurons of LPS-treated Nurr1^cKO^ mice compared to the LPS-treated control mice or PBS-injected Nurr1^cKO^ mice (Fig. 4D, E). Overall, our findings suggest that Nurr1 deficiency in microglia exacerbates LPS-induced DAergic neurodegeneration and increases the level of phosphorylated-α-synuclein, underscoring the critical role of microglial Nurr1 in the pathogenesis of PD.

### Nurr1 deficiency exacerbates microglial activation, pro-inflammatory responses and NLRP3 inflammasome activation in vivo

To elucidate the pathological processes initiated in Nurr1-knockdown microglia that contribute to neurodegeneration, we initially examined the activation of microglia in the midbrain of Nurr1^cKO^ and control mice. As depicted in Additional file 1: Figure S2A and S2B, we observed a 31% increase in the number of microglia in the SNc of 12-month-old Nurr1^cKO^ mice (*p* < 0.05), as determined by IF staining of microglia marker Iba1. Furthermore, detailed morphological analysis revealed that microglia lacking Nurr1 displayed thickened and retracted branches, along with enlarged cell bodies, as quantified by assessing the microglial soma size (*p* < 0.01, Additional file 1: Figure S2C) and process occupied area (*p* < 0.01, Additional file 1: Figure S2D). These findings provide evidence that Nurr1 deficiency triggers a shift in microglial polarization toward an activated state.

To determine whether Nurr1 deficiency could contribute to the microglia shift from a homeostatic towards a ‘disease-associated’ phenotype, we analyzed different molecular signatures of microglia in the Nurr1^cKO^ and control mice. As shown in Additional file 1: Figure S2E and S2G, a substantial fraction of microglia in the Nurr1^cKO^ mice were positive for CD68 (*p* < 0.01), a disease-associated microglia (DAM) marker of activated cells. In contrast, CD206, as an immunosuppressive phenotype marker of microglia, was rarely co-localized in the Iba1-positive microglia of Nurr1^cKO^ mice (*p* < 0.01, Additional file 1: Figure S2F and S2H). Meanwhile, we also quantified the mRNA levels of DAM genes, including Cx3cr1, Itgax, Trem2, and Tmem119, showing increased levels of Trem2 and Itgax (*p* < 0.0001) and decreased levels of Cx3cr1 and Tmem119 (*p* < 0.05) in the midbrain of the 12-month-old Nurr1^cKO^ (Additional file 1: Figure S2I). Collectively, these data indicate that Nurr1 deficiency may shift microglial activation towards a DAM phenotype primed for increased neurotoxicity.

To investigate the potential impact of Nurr1 deficiency on miR-30e-5p expression in vivo, we assessed the level of miR-30e-5p in both PBMCs and primary microglia of Nurr1^cKO^ and control mice. Through qRT-PCR analysis, we observed significantly decreased miR-30e-5p expression level in PBMCs (*p* < 0.05) and primary microglia (*p* < 0.05) of Nurr1^cKO^ compared to the controls** (**Additional file 1: Figure S3). These findings strongly suggest that Nurr1 may influence miR-30e-5p expression in vivo, which is supported by the consistent results observed in PBMCs samples from PD patients.

**Fig. 5:**
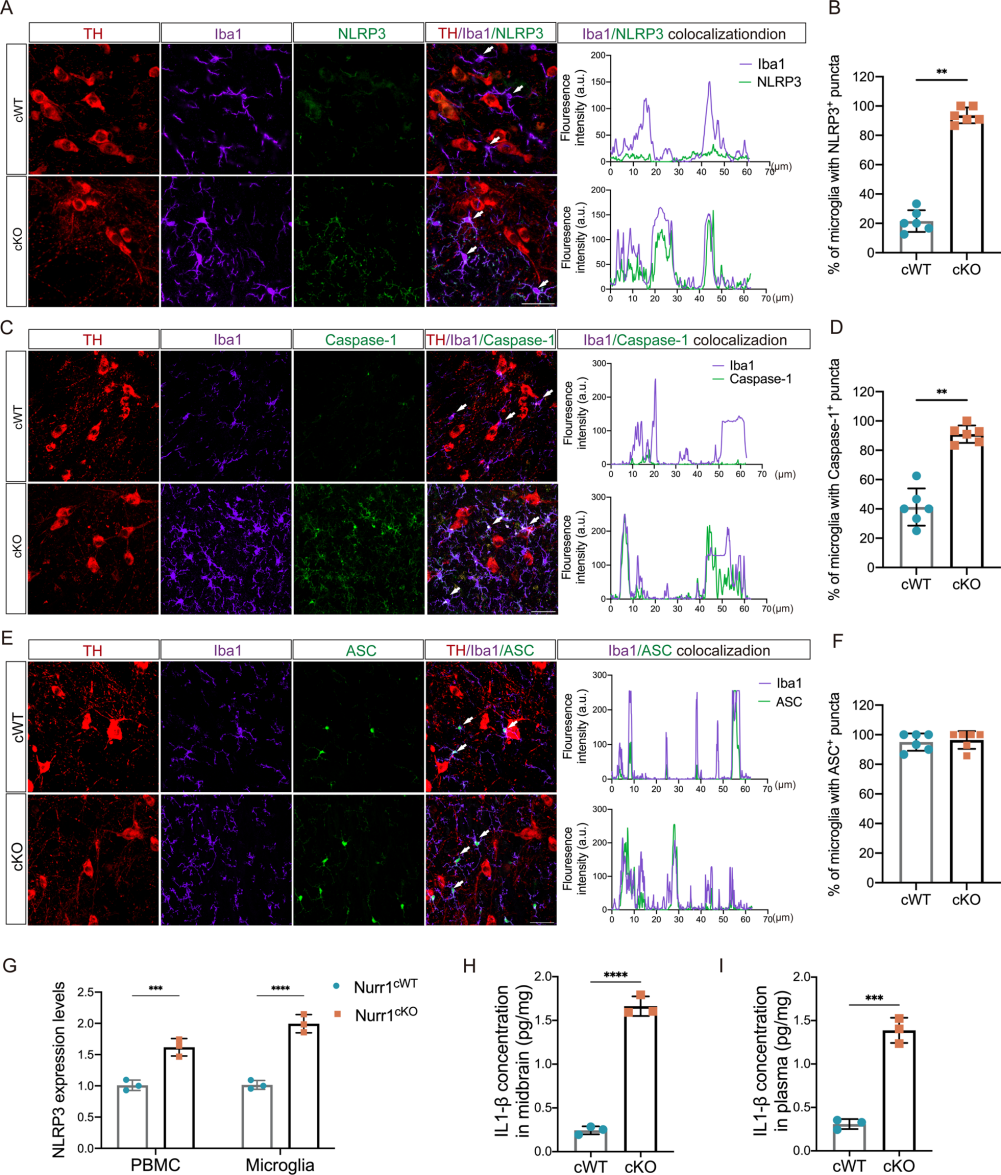
NLRP3 inflammasome is activated in the microglia of Nurr1^cKO^ mice. **A** Representative images with TH (red), Iba-1 (purple) and NLRP3 (green) immunostaining and the colocalization analyses of Iba-1 and NLRP3 in SNc of 12-month-old Nurr1^cKO^ mice and Nurr1^cWT^ mice. Scale bar = 20 μm. **B** The proportion of Iba1-positive and NLRP3-positive microglia in the SNc was quantified. Data are presented as the means ± SD. Unpaired Student T test, **p < 0.01. **C** Representative images with TH (red), Iba-1 (purple) and Caspase-1 (green) immunostaining and the colocalization analyses of Iba-1 and Caspase-1 in SNc of the mice. Scale bar = 20 μm. **D** Quantification of the proportion of Iba1-positive and Caspase-1-positive microglia in the SNc of Nurr1^cKO^ and Nurr1^cWT^ mice. Data are presented as the means ± SD. Unpaired Student T test, **p < 0.01. **E** Triple-label immunofluorescence of TH (red), Iba-1 (purple) and ASC (green) in SNc of the mice. Scale bar = 20 μm. **F** The proportion of Iba1-positive and ASC-positive microglia in the SNc was quantified. n = 3 mice per group. **G** The expression level of NLRP3 in PBMCs and microglia of Nurr1^cKO^ and Nurr1^cWT^. **H** The level of IL-1β in the midbrain of Nurr1^cKO^ and Nurr1^cWT^ mice. **I** The level of IL-1β in the plasma of Nurr1^cKO^ and Nurr1^cWT^ mice. Data are presented as the means ± SD. **p < 0.01, ***p < 0.001. Unpaired Student T test

We then investigated whether Nurr1 deficiency exacerbates NLRP3 inflammasome activation in microglia in vivo. Triple-staining of Iba1, NLRP3 and TH showed that microglia in the SNc of Nurr1^cKO^ mice displayed concentrated NLRP3 puncta, while the NLRP3 activation was barely observed in Nurr1^cWT^ mice (*p* < 0.01, Fig. 5A, B). Correspondingly, the amount of Caspase-1 co-localized with Iba1 is higher in the Nurr1^cKO^ mice than in control mice (*p* < 0.01, Fig. 5C, D). However, no significant difference in ASC immunoreactivity was found in the microglia between Nurr1^cKO^ and Nurr1^cWT^ mice (Fig. 5E, F). To further validate these results, we also assessed the expression of NLRP3 mRNA level in the PBMCs and primary microglia obtained from Nurr1^cko^ and Nurr1^cWT^ mice. Our results demonstrated a significant increase in the expression level of NLRP3 in both PBMCs and primary microglia of Nurr1^cko^ mice when compared to the controls (*p* < 0.001, Fig. 5G). Furthermore, ELISA was performed to evaluate the concentrations of the inflammatory cytokine IL-1β in the midbrain and plasma caused by Nurr1 deficiency. As illustrated in Fig. 5H and I, the absence of Nurr1 in Cd111b-positive cells resulted in a notable rise in IL-1β level compared to those observed in the midbrain and plasma of control mice (*p* < 0.001). Taken together, these results indicate that the NLRP3 inflammasome is activated and involved in the microglial activation caused by Nurr1 deficiency.

### Nurr1 promotes the transcription of the precursor miR-30e-5p

In order to gain further insight into the molecular mechanism of interactions among Nurr1, miR-30e-5p and NLRP3 in vitro, we first set up an investigation to evaluate whether Nurr1 directly binds with NLRP3. To achieve this, we established a stable Nurr1 knockdown BV2 microglia cell line (ShNurr1-BV2) and confirmed the successful knockdown of Nurr1 through RT-qPCR and Western blotting (Additional file 1: Figure S4A, S4B). Notably, both NLRP3 protein and mRNA levels were elevated in ShNurr1-BV2 cells compared to the control cells (Additional file 1: Figure S4A, S4B), indicating that Nurr1 may exert a transcriptional effect on the mRNA level of NLRP3. However, our bioinformatics prediction using the JASPAR database (http://jaspar.genereg.net/) did not identify any potential transcription factor binding sites (TFBS) of Nurr1 within the 2000 bp upstream promotor region of the NLRP3 gene. Consequently, we hypothesized that Nurr1 may regulate the mRNA level of NLRP3 indirectly to exert its anti-inflammatory effect in microglia.

**Fig. 6:**
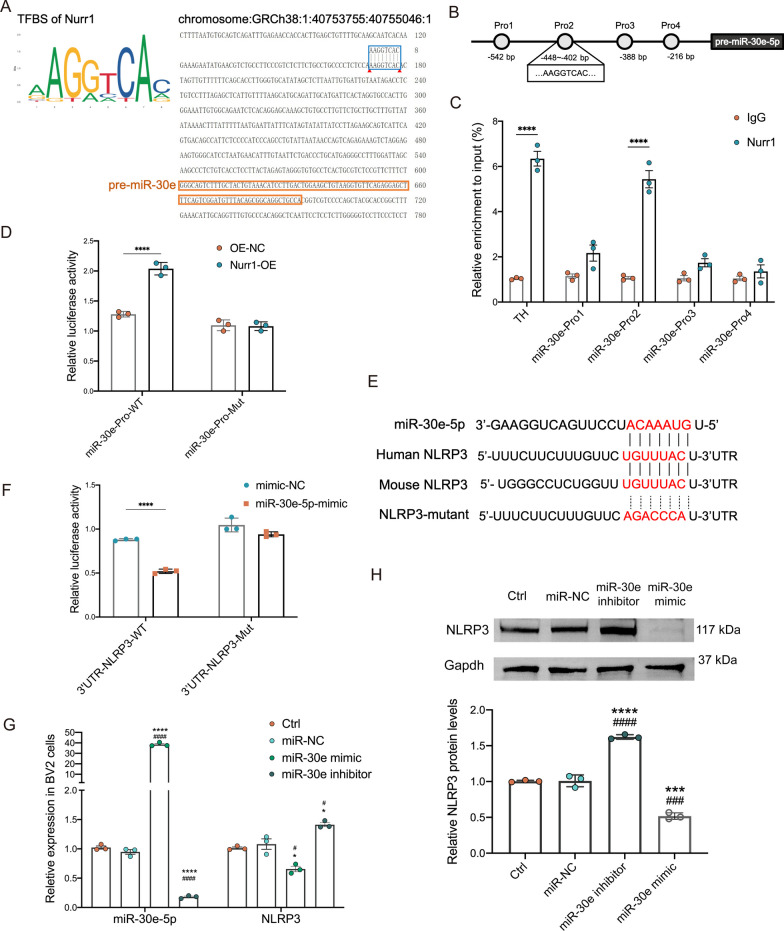
Nurr1 promotes the transcription of pre-miR-30e-5p, and the targeting relationship between miR-30e-5p and NLRP3. **A** Schematic representation illustrating the binding region of Nurr1 specific to the promoter of pre-miR-30e-5p. Nurr1 transcription factor binding sites were identified within 404–448 bp upstream of pre-miR-30e-5p in mice. **B** ChIP-PCR assay conducted in BV2 cells using specific primers for the promoter regions (Pro1, − 448 to − 542 bp; Pro2, − 402 to − 448 bp; Pro3, − 388 to − 402 bp; Pro4, − 216 to − 388 bp) of miR-30e-5p. **C** Recruitment of Nurr1 to the Pro2 region was significantly enhanced compared to the IgG group. ****p < 0.0001, Unpaired Student T test. **D** A dual luciferase reporter assay was employed to detect Nurr1’s transcription activity with miR-30e-5p. ****p < 0.0001, Two-way ANOVA Tukey test. **E** Schematic diagram of binding sites between miR-30e-5p, human and mouse NLRP3 3ʹUTR predicted by bioinformatics databases. **F** The targeting relationship between miR-30e-5p and NLRP3 was verified using a dual-luciferase reporter gene assay in 293T cells. 3ʹUTR-NLRP3-WT refers to NLRP3 wild type 3’UTR and 3ʹUTR- NLRP3-Mut refers to NLRP3 mutant 3ʹUTR. ****p < 0.0001, Two-way ANOVA Tukey test. **G** The BV2 cells were treated with miR-30e-5p mimic, miR-30e-5p inhibitor and miR-NC for 24 h. Representative immunoblots and quantitative analysis of NLRP3 protein level (**H**), and the relative expression level of NLRP3 mRNA (**G**) are shown. Gapdh was used as a loading control. **G** The transfection efficiency of miR-30e-5p mimic and miR-30e-5p inhibitor in BV2 cells. *, *p* < 0.05, ***, *p* < 0.001; ****, *p* < 0.0001, relative to the blank BV-2 cells (Ctrl); ^#^, *p* < 0.05, ^###^, *p* < 0.001; ^####^, *p* < 0.0001, relative to the miR-NC. One-way ANOVA Tukey test. These experiments were repeated three times independently

We initially showed that Nurr1 knockdown reduced the expression of miR-30e-5p in RAW264.7 macrophage cells (*p* < 0.0001, Fig. 1D), and our subsequent objective was to investigate whether Nurr1 governs the transcription of the miR-30e-5p precursor (pre-miR-30e-5p). To address this, we started a bioinformatics analysis employing the JASPAR database for predictive insights. Within approximately 430 bp upstream of the pre-miR-30e-5p in mice, eight TFBS of Nurr1 were identified (Fig. 6A). To confirm the predicted TFBS of Nurr1 in the promoter region of the miR-30e-5p gene, we performed a ChIP assay using a Nurr1-specific antibody in BV2 cells. Figure 6B illustrates the specific primers designed for the promoter regions (Pro1, − 448 to − 542 bp; Pro2, − 402 to − 448 bp; Pro3, − 388 to − 402 bp; Pro4, − 216 to − 388 bp) within the pre-miR-30e-5p. The analysis of epidermal lysates demonstrated a significant increase in the recruitment of Nurr1 to Pro2 within the miR-30e-5p gene (*p* < 0.0001, Fig. 6C). To validate these findings, we utilized TFBS located in the promoter region of the TH gene, a well-established Nurr1 target gene, as a positive control. These observations provided strong evidence that Nurr1 specifically binds to the Pro2 site in the miR-30e-5p promotor.

Moreover, to investigate the specificity of Nurr1 in the transcriptional regulation of miR-30e-5p, we conducted a dual-luciferase reporter assay. In this assay, we cloned the predicted target region (Pro2) and the mutated sequence of the pre-miR-30e-5p into the luciferase reporter plasmids (miR-30e-pro-WT/miR-30e-pro-Mut). Subsequently, the firefly luciferase reporter constructs and the control pRL-TK plasmid were co-transfected into 293T cells with either Nurr1 overexpression (Nurr1-OE) or the vector control (OE-NC). The Renilla luciferase activity was used to normalize the firefly luciferase activity of each sample. Our findings demonstrated that the overexpression of Nurr1 significantly enhanced the relative luciferase activity of miR-30e-pro-WT (*p* < 0.0001, Fig. 6D). Furthermore, the miR-30e-pro-Mut construct disrupted the binding of Nurr1 to the promoter region of the pre-miR-30e-5p (Fig. 6D). Taken together, these results strongly suggest that the upregulation of Nurr1 function promotes the transcription of pre-miR-30e-5p.

### MiR-30e-5p regulates the expression of NLRP3 gene

Given that miRNAs predominantly exert their biological functions by inhibiting target mRNAs through binding to their 3'UTR regions, our subsequent aim was to assess whether miR-30e-5p targets the NLRP3 gene. To achieve this, we utilized online prediction databases TargetScan and miRbase (www.targetscan.org and www.mirbase.org). Through bioinformatics analysis, we discovered seven binding sites within the ‘seed’ sequence of miR-30e-5p that were predicted to match the 3ʹUTR of both human and mouse NLRP3 mRNA (Fig. 6E), suggesting NLRP3 as a promising candidate target for miR-30e-5p.

To confirm NLRP3 as a direct target of miR-30e-5p, we cloned the wild-type sequence of NLRP3’s 3′UTR (3′UTR-NLRP3-WT) and a mutant sequence (3′UTR-NLRP3-Mut) into a dual-luciferase reporter gene vector for conducting the luciferase assay. As depicted in Fig. 6F, transfection of the miR-30e-5p mimic (20 ng/ml) resulted in a reduction in luciferase activity associated with the 3′UTR-NLRP3-WT plasmid (*p* < 0.0001). In contrast, transfection of the 3′UTR-NLRP3-Mut plasmids did not alter luciferase activity in the presence of the miR-30e-5p mimic and showed comparable results to the 3′UTR-NLRP3-WT.

Next, we assessed the impact of miR-30e-5p on NLRP3 expression level in microglia through transient transfection with miR-30e-5p mimic, miR-30e-5p inhibitor, and miRNA-negative control (miR-NC) for 24 h. Figure 6H illustrates the transfection efficiency of miR-30e-5p. As anticipated, the transfection of BV2 cells with miR-30e-5p mimics significantly reduced both NLRP3 mRNA and protein levels (*p* < 0.001, Fig. 6G, H). Conversely, BV2 cells transfected with miR-30e-5p inhibitor exhibited an upregulation of NLRP3 when compared to cells transfected with miR-NC (*p* < 0.0001, Fig. 6G, H). In conclusion, these findings indicate that NLRP3 is indeed the genuine endogenous target of miR-30e-5p in microglia.

### Nurr1 deficiency up-regulates the expression of NLRP3 by attenuating the expression of miR-30e-5p in vitro

**Fig. 7:**
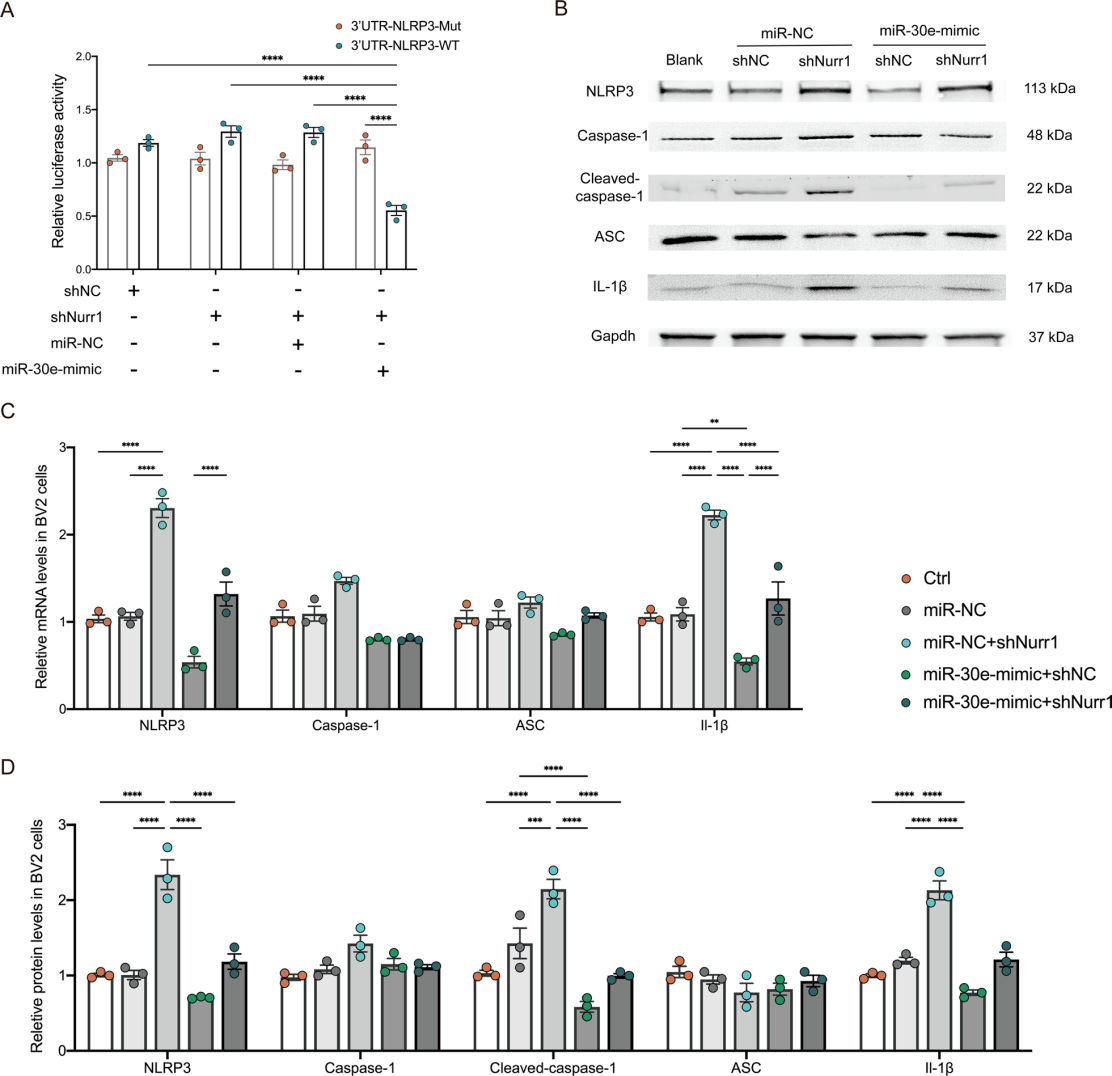
Nurr1 deficiency upregulates NLRP3 expression by attenuating miR-30e-5p expression. **A** Relative luciferase activity of shNurr1-BV2 cells transfected with 3ʹUTR-NLRP3-WT or 3ʹUTR-NLRP3-Mut, along with miR-NC or miR-30e-mimic. ****p < 0.0001, Two-way ANOVA Tukey test. **B** ShNurr1-BV2 and ShNC-BV2 cells treated with miR-30e-5p mimic or miR-NC, respectively. Representative immunoblots (**B**) and quantitative analysis of NLRP3, Caspase-1, Cleaved-caspase-1, ASC, and IL-1β protein levels (**D**) and relative mRNA levels of NLRP3, Caspase-1, ASC, and IL-1β (**C**) are shown. Gapdh was used as a loading control. Data are presented as mean ± SD. **p < 0.01, ***p < 0.001, and ****p < 0.0001, One-way ANOVA Tukey test

To further demonstrate that the increased expression of the NLRP3 gene in Nurr1-depleted microglia is a result of a decreased miR-30e-5p expression, we initially transfected the 3′UTR reporter plasmids for NLRP3 (containing the miR-30e-5p seed sequence) into shNurr1-BV2 and shNC-BV2 cells, followed by an analysis of luciferase activity in cell extracts. Notably, the upregulated expression of miR-30e-5p in shNurr1-BV2 cells significantly suppressed the luciferase activity of the predicted targets (*p* < 0.0001, Fig. 7A). Subsequently, we examined the effects of Nurr1 on NLRP3 expression in the presence or absence of miR-30e-5p. ShNurr1-BV2 cells, which exhibited a reduced level of miR-30e-5p (Additional file 1: Figure S4B), displayed a significantly higher level of NLRP3 (*p* < 0.0001, Fig. 7B–D, Additional file 1: Figure S4B). Moreover, the elevated protein and mRNA level of NLRP3 observed upon Nurr1 knockdown were rescued upon transfection of a specific miR-30e-5p mimic into ShNurr1-BV2 cells (Fig. 7B–D). Consistently, the increased protein levels of Cleaved-caspase-1 and IL-1β (Fig. 7B, D), as well as the mRNA levels of Caspase-1 and IL-1β (Fig. 7C), were detected in ShNurr1-BV2 cells and were then reversed by miR-30e-5p overexpression (*p* < 0.0001), indicating the activation of NLRP3 inflammasome. Overall, these findings provide compelling evidence that Nurr1 regulates NLRP3 expression through miR-30e-5p in microglia.

## Discussion

The main pathological hallmark of PD is the selective degeneration of DAergic neurons in the SN [1]. The development and maintenance of these neurons are regulated by unique sets of transcription factors, among which Nurr1 has been implicated in the pathogenesis of PD [17]. Moreover, the midbrain exhibits a high abundance of microglia, which may render DAergic neurons in this region more susceptible to inflammation [27]. This study demonstrated the pivotal role of Nurr1 as a regulator of neuroprotective responses, exerting control over the miR-30e-5p-NLRP3-IL-1β pathway. In line with the in vivo findings, positive correlations were also observed among the *NURR1*, miR-30e-5p, *NLRP3*, and IL-1β levels in the PBMC of a relatively larger sample size comprising PD patients and HC cohorts. Furthermore, our in vitro studies provide compelling evidence of the molecular interactions among Nurr1, miR-30e-5p and NLRP3. These findings suggest the importance of Nurr1-miR-30e-5p-NLRP3 axis in regulating inflammation and neuronal survival in PD, and provide new insights into the disease pathogenesis, highlighting potential biomarkers and therapeutic targets for PD.

By establishing the Nurr1^cKO^ mouse model, we emphasized the crucial role of Nurr1 in the microglia-mediated inflammatory reaction and the cross-talk between microglia and DAergic neurons. Our study results align with our previous research [12], demonstrating that conditional knockout of Nurr1 in microglia leads to a reduction in striatal DA level, a decrease in DAergic neuron number, and an increased aggregation of α-synuclein following systemic LPS stimulation. Consistent with our findings, Saijo et al. observed a significant decrease in DAergic neurons in the SNc of shNurr1 lentivirus-injected mice compared to control mice after LPS treatment [11]. They elucidated that Nurr1 exerts anti-inflammatory effects by recruiting CoREST corepressor complexes to NF-κB target inflammatory genes, thereby inhibiting the expression of inflammatory mediators in microglia [11]. Previous studies have extensively demonstrated the neurotoxicity induced by inflammatory factors [28]. It is worth noting that microglial activation is a double-edged sword, exerting both detrimental and beneficial effects on the neurons by producing pro-inflammatory or immunosuppressive cytokines, depending on the functional phenotypes of microglia [3]. In our Nurr1^cKO^ mouse model, we observed an increased number of microglia with enhanced reactivity, polarizing from a homeostatic to an activated state. Moreover, Nurr1 deficiency resulted in a significant proportion of DAM-like phenotype microglia, which is a recently identified subpopulation characteristically associated with neurodegenerative diseases, as determined by the changes of Cx3cr1, Itgax, Trem2, and Tmem119 [29]. The transition to a DAM phenotype is thought to provide microglia with key neuroprotective capabilities early in disease progression. However, DAM becomes dysregulated at later stages and accelerates neurodegeneration [29, 30]. This finding aligns with our previous discovery of elevated pro-inflammatory factors IL-1β and TNF-α levels, along with decreased TGF-β level in the midbrain of 17-month-old Nurr1^cKO^ mice, indicating the detrimental role of DAM presence in later stages of neurodegeneration [12]. Overall, we hypothesize that under conditions of Nurr1 deficiency, the survival rate of DAergic neurons decreases in response to inflammatory stimuli due to the elevated levels of pro-inflammatory cytokines mediated by the DAM phenotype of microglia.

NLRP3 inflammasome is the most characterized inflammasome known to activate the innate immune response and induces the release of potent pro-inflammatory cytokines IL-1β and IL-18, which are believed to contribute to neurodegeneration [16]. Our study observed elevated levels of PBMCs *NLRP3* and plasma IL-1β in PD patients compared to HC. These findings are consistent with previous studies that reported increased NLRP3 inflammasome activity and release of inflammatory cytokines in PD patients [31, 32]. Furthermore, multiple studies have demonstrated the involvement of NLRP3 inflammasome in the degeneration of DAergic neurons [16, 33]. It has been shown that pathological α-synuclein can trigger the transcription of NLRP3 through binding to TLR2, leading to its activation upon phagocytosis of aggregated α-synuclein by microglia [34]. The activated NLRP3 inflammasome accelerates the production of inflammatory cytokines and reactive oxygen species, ultimately contributing to neuronal injury [34]. Despite NLRP3 inhibitors having entered clinical trials for the treatment of PD, there is currently insufficient understanding of the effector mechanisms that trigger NLRP3 transcription and influence disease pathogenesis. Our in vivo data has unveiled that NLRP3 inflammasome is activated and involved in the microglial activation caused by Nurr1 deficiency, highlighting a novel endogenous Nurr1-related mechanism underlying the initiation of NLRP3 inflammasome activity in microglia.

With the advancement in deep sequencing technologies, more miRNAs have been discovered, and their significant roles in PD have also been corroborated [18]. In this study, we employed miRNA-seq, qRT-PCR, and bioinformatics analyses and identified differentially expressed miRNAs, namely miR-21-5p, miR-30e-5p, miR-29a-5p, and miR-105-5p, in PD patients, all of which are associated with neuroinflammatory pathways. Notably, miR-30e-5p exhibited a marked reduction under Nurr1 deficiency conditions. Furthermore, we validated the decreased level of miR-30e-5p in a relatively large sample size of PD patients, and these levels displayed negative correlations with disease duration and motor severity. To the best of our knowledge, the dysregulation of miR-30e-5p in PD patients has not been reported. However, previous studies have shown reduced expression of miR-30e in the SNc of an MPTP-induced PD mouse model, and other members of the miR-30 family have been implicated in PD pathogenesis [35, 36]. For example, miR-30b has been identified as dysregulated in the SN of PD patients compared to healthy controls in post-mortem human brain studies [37], and differential expression of miR-30b and miR-30c has been observed in PBMCs of PD patients compared to healthy controls [35]. Our findings further establish the targeting relationship between miR-30e-5p and NLRP3, where NLRP3 mRNA level are altered upon miR-30e-5p overexpression. This suggests that miR-30e-5p may accelerate the degradation of NLRP3 mRNA by binding to specific sites in the 3′UTR of NLRP3, as demonstrated in microglia and RAW 264.7 macrophage cells [26]. Consistent with our data, inhibition of miR-30e resulted in increased expression of NLRP3, cleaved caspase-1, and IL-1β [26]. Taken together, our results indicate that a Nurr1-dependent signature miRNA miR-30e-5p plays a pivotal role in microglia-mediated inflammation by modulating the expression of NLRP3. Future investigations into the role of miR-30e-5p and its target NLRP3 in vivo models are needed to further elucidate its therapeutic applicability.

The transcription factor and miRNA regulatory network are defined by the effects of the transcription factor on the target gene and post-transcription interactions between miRNA and their target genes [38]. While previous studies have shown that Nurr1 regulates cellular RNA composition through its effect on signal transduction, transcription, and post-transcriptional processes [17], the specific miRNA modulated by Nurr1 has not been thoroughly examined. This study demonstrated the targeting relationships between Nurr1 and miR-30e-5p using ChIP and dual-luciferase reporter assays. Additionally, our data indicate that the increased expression of the NLRP3 in Nurr1-depleted microglia is dependent on miR-30e-5p. Notably, rescue experiments demonstrated that overexpression of miR-30e-5p partially reversed the elevated NLRP3 level caused by Nurr1 deficiency. These findings suggest that Nurr1 may modulate the expression of NLRP3, at least in part, through transcriptional regulation of mi-30e-5p. Furthermore, statistical analyses in this study showed significant associations among levels of *NURR1*, miR-30e-5p and *NLRP3* in the PBMCs of PD patients, which verifies the existence of the Nurr1-miR-30e-5p-NLRP3 axis in peripheral immunity cells. Considering culturing isolated human microglia ex vivo is challenging due to its restricted proliferative capacity, cell viability, and rapid changes to its unique CNS identity once removed from the brain microenvironment [39]. PBMC signatures have been shown to reflect the disease-related molecular changes in the brain and were widely used to identify potential biomarkers for PD [40, 41]. Indeed, the ROC curve analyses showed that the combination of PBMCs *NURR1*, miR-30e-5p and *NLRP3* enhances the discriminatory accuracy between PD and HC, indicating that the combination of *NURR1*, miR-30e-5p and *NLRP3* expression in PBMCs could be utilized as collective biomarkers for PD diagnosis.

In conclusion, our study provides a novel insight into the role of Nurr1 in inflammation associated with PD. Through miRNA-sequencing and verification in a relatively larger sample size cohort, we identified miR-30e-5p as a Nurr1-dependent miRNA, the change of which may distinguish PD patients from HC. Furthermore, we identified correlations among the expression levels of *NURR1*, miR-30e-5p, and *NLRP3* in the PBMCs and the plasma concentration of IL-1β in patients with PD. Our investigation into the role of Nurr1 in inflammation-mediated PD pathology, using Nurr1^cKO^ mice, provided compelling evidence that the absence of PBMCs and microglial Nurr1 leads to significant degeneration of DAergic neurons and aggregation of α-synuclein in response to inflammation. Additionally, the reduced level of miR-30e-5p and heightened activation of the NLRP3 inflammasome further demonstrated the relationships between Nurr1-miR-30e-5p-NLRP3 in vivo. In vitro experiments solidified these findings by revealing that dysfunctional Nurr1 upregulates NLRP3 expression by suppressing miR-30e-5p. Taken together, our results shed light on the pivotal contribution of the Nurr1-miR-30e-5p-NLRP3 axis to inflammation in PD. Moreover, our investigations in PD patients reinforce the clinical relevance of these findings, suggesting the potential utility of these biomarkers and providing a solid foundation for the development of targeted therapeutic interventions.

### Supplementary Information

**Additional file 1: Table S1.** List of primary antibodies for Western blotting (WB) and Immunofluorescent staining (IF). **Table S2.** List of primers used for quantitative real-time PCR assays. **Figure S1.** Conditional Nurr1 deletion inCd11b-expressingPBMCand microglia. **Figure S2.** Nurr1 deficiency in microglia aggravates pro-inflammatory responses. **Figure S3.** Expression level of miR-30e-5p in PBMCs and microglia of Nurr1cKO and Nurr1cWT. **Figure S4.** Stable Nurr1 knockdown in BV2 microglia.

## Data Availability

The data supporting this study’s findings are available from the corresponding author upon reasonable request.
