# Supplementary material for: The role of Nurr1-miR-30e-5p-NLRP3 axis in inflammation-mediated neurodegeneration: insights from mouse models and patients’ studies in Parkinson’s disease
Source: J Neuroinflammation. 2023 Nov 22;20:274. doi: 10.1186/s12974-023-02956-x (PMC10664369; doi:10.1186/s12974-023-02956-x)
Supplement: Supplementary file 1 — Additional file 1: Table S1. List of primary antibodies for Western blotting (WB) and Immunofluorescent staining (IF). Table S2. List of primers used for quantitative real-time PCR assays. Figure S1. Conditional Nurr1 deletion inCd11b-expressingPBMCand microglia. Figure S2. Nurr1 deficiency in microglia aggravates pro-inflammatory responses. Figure S3. Expression level of miR-30e-5p in PBMCs and microglia of Nurr1cKO and Nurr1cWT. Figure S4. Stable Nurr1 knockdown in BV2 microglia. [file 12974_2023_2956_MOESM1_ESM.pdf]

Supplementary materials

Table S1. List of primary antibodies for Western blotting (WB) and Immunofluorescent staining (IF).

**Antibody**	**Company**	**Hosts**	**Catalog number**	**Application**	**Dilution**
Anti-TH	Millipore	Rabbit	AB152	WB	1:1000
Anti-GAPDH	Cell Signaling Technology	Rabbit	2118S	WB	1:4000
Anti-Nurr1	Abcam	Rabbit	Ab176184	WB	1:500
Anti-NLRP3	Cell Signaling Technology	Rabbit	15101	WB	1:1000
Anti-Cleaved-caspase-1	Cell Signaling Technology	Rabbit	89332	WB	1:1000
Anti-ASC	Cell Signaling Technology	Rabbit	67824	WB	1:1000
Anti-IL-1β	Cell Signaling Technology	Rabbit	31202	WB	1:1000
Anti-TH	Millipore	Chicken	AB9702	IF	1:500
Anti-α-synuclein (phospho S129)	Abcam	Rabbit	Ab51253	IF	1:500
Anti-Iba1	FUJIFILM Wako	Rabbit	019-19741	IF	1:1000
Anti-Nurr1	Proteintech	Rabbit	10975-2-AP	IF	1:200
Anti-CD68	Arigo	Mouse	ARG10514	IF	1:200
Anti-CD206	Arigo	Mouse	ARG55554	IF	1:200

Anti-NLRP3	Adipogen	Mouse	AG-20B-0014	IF	1:200
Anti-Caspase-1	Santa Cruz	Mouse	Sc-56036	IF	1:500
Anti-ASC	Adipogen	Mouse	AG-25B-0006-C100	IF	1:500

Table S2. List of primers used for quantitative real-time PCR assays.

mRNA	Primers
NLRP3 (human)	Forward: 5’-GTTTGACCCCGATGATGAGC- 3’
	Reverse: 5’-CTTGTGGATGGGTGGGTTTG- 3’
GAPDH (human)	Forward: 5’-GCGGTCACGTTTTCCACTATG- 3’
	Reverse: 5’-GAAGATGGTGATGGGATTTC- 3’
Nurr1-flox	Forward: 5’-AAACAAAACAGGGCAACAGG- 3’
	Reverse: 5’-GCCTGTGCTGTAGTTGTCCA- 3’
Cd11b-cre	Forward: 5’-CAGGTATGCTCAGAAAACGCCT- 3’
	Reverse: 5’-TGGGCCAACCCAAGAAACAAGT- 3’
Nurr1 (mouse)	Forward: 5’-ATTCCAGGTTCCAGGCAAAC - 3’
	Reverse: 5’-AGCAAAGCCAGGGATCTTCT - 3’
Gapdh (mouse)	Forward: 5’-GCATTGTGGAAGGGCTCATG - 3’
	Reverse: 5’-AGGGATGATGTTCTGGGCAG - 3’
NLRP3 (mouse)	Forward: 5’-TCTCCCGCATCTCCATTTGT - 3’
	Reverse: 5’-CTGTCCCGCATTTTAGTCCG - 3’
IL-1β (mouse)	Forward: 5’- TTCTTTTCCTTCATCTTTGAAGAAG- 3’
	Reverse: 5’- TCCATCTTCTTCTTTGGGTATTGTT- 3’
ASC (mouse)	Forward: 5’-CTCTGTATGGCAATGTGCTGAC- 3’
	Reverse: 5’-GAACAAGTTCTTGCAGGTCAG- 3’
Caspase-1 (mouse)	Forward: 5’-ACAAGGCACGGGACCTATG- 3’

	Reverse: 5'- TCCCAGTCAGTCCTGGAAATG- 3'

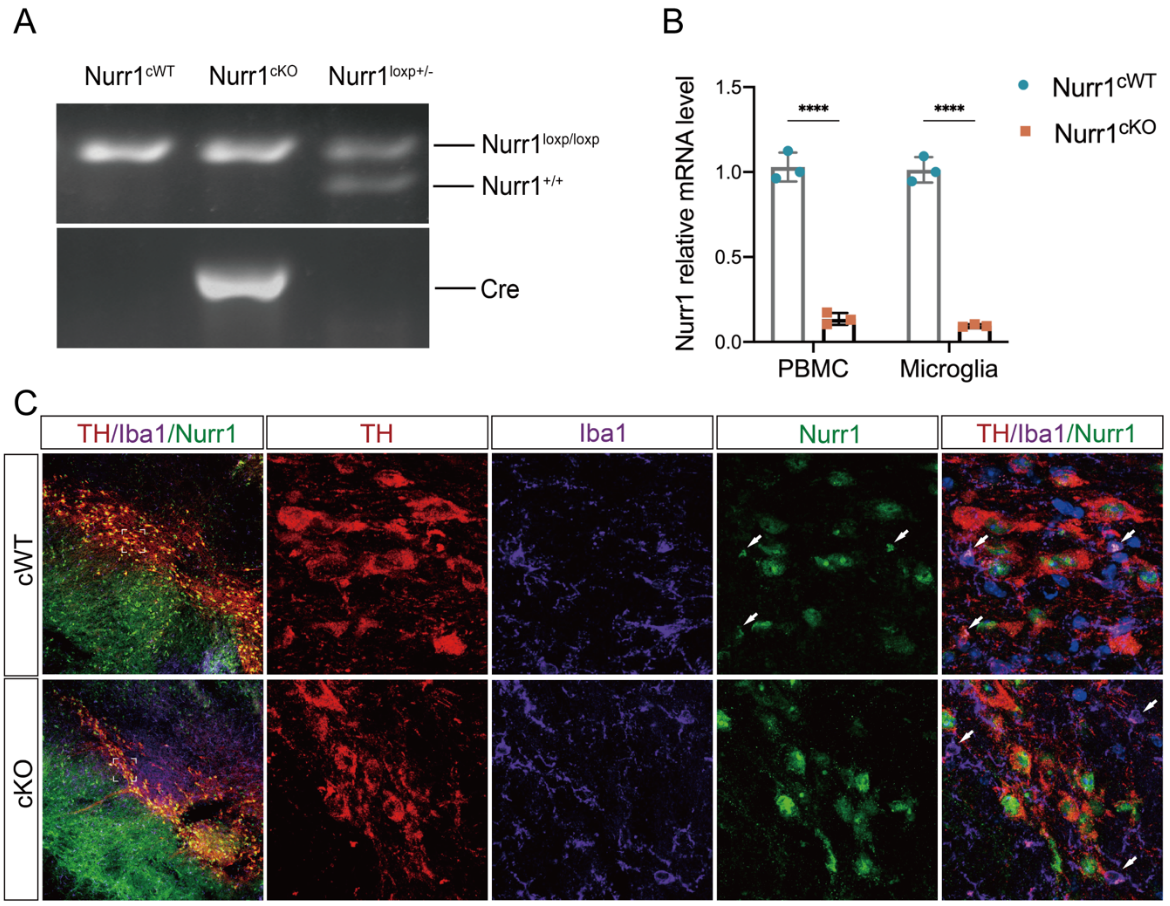

Supplementary Figure S1. Conditional Nurr1 deletion in Cd11b-expressing PBMC and microglia. **(A)** Tail genotyping of Nurr1 floxed allele (upper) and Cd11b-Cre transgene (lower). **(B)** qRT-PCR analysis of Nurr1 mRNA in PBMC and primary microglia in Nurr1^{cKO} and Nurr1^{cWT} (n = 3 per each group). Unpaired t test: ***p< 0.001. **(C)** IFC analysis for Nurr1 expression in microglia was performed using an antibody against Nurr1 (green) together with TH (red) in 2-month-old Nurr1^{cKO} and Nurr1^{cWT} mice (scale bar: 100 µm; high-magnification, 25 µm).

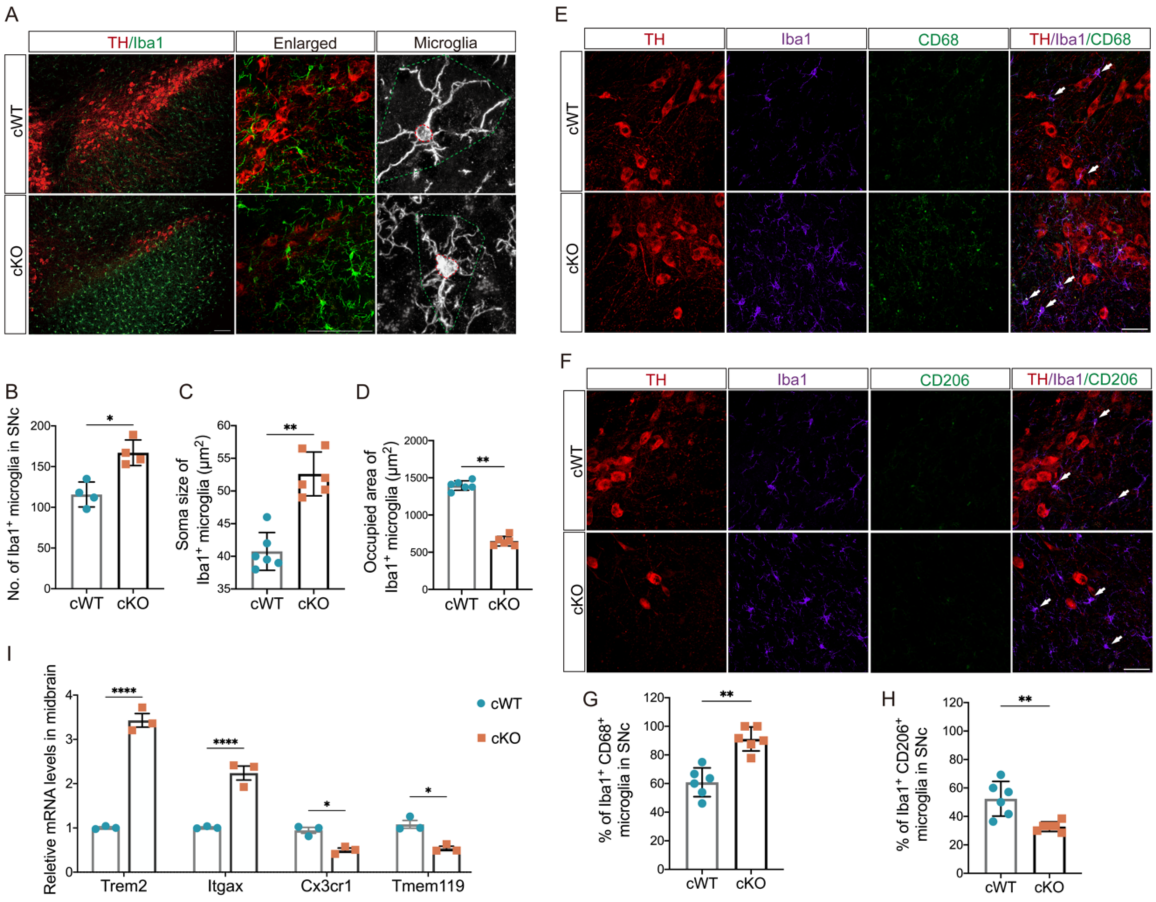

Supplementary Figure S2. Nurr1 deficiency in microglia aggravates pro-inflammatory responses. (A) Double-label immunofluorescence of Iba-1 (green) and TH (red) in SNc in the 12-month-old Nurr1^{cKO} and Nurr1^{cWT} mice. Representative images of microglia from Nurr1^{cKO} and Nurr1^{cWT} mice showing measurement of soma size (outlined by red line) and occupied area (green line). Scale bars: 20 mm. **(B)** Number of Iba-1 positive microglia in SN of 12-month-old Nurr1^{cKO} and Nurr1^{cWT} mice. Quantification of the microglial process soma size **(C)** and occupied area **(D)** in SN of Nurr1^{cKO} and Nurr1^{cWT} mice (n = 3 mice per genotype). **(E)** Triple-label immunofluorescence of TH (red), Iba-1 (purple) and CD68 (green) in SNc of the 12-month-old Nurr1^{cKO} and Nurr1^{cWT} mice. Scale bar = 20 μm. **(F)** Triple-label immunofluorescence of TH (red), Iba-1 (purple) and CD206 (green) in SNc of the 12-month-old Nurr1^{cKO} and Nurr1^{cWT} mice. Scale bar = 20 μm. **(G)** Quantification of the proportion of Iba1-positive and CD68-positive microglia in the SNc of Nurr1^{cKO} and Nurr1^{cWT} mice. **(H)** Quantification of the proportion of Iba1-positive and CD206-positive microglia in the SNc of Nurr1^{cKO} and Nurr1^{cWT} mice (n = 3 mice per genotype). **(I)** Expression levels of DAM mRNAs in the midbrain of Nurr1^{cKO} and control mice. Data are presented as the means ± SD. Unpaired Student T test, *$p < 0.05$, **$p < 0.01$, ***$p < 0.001$, ****$p < 0.0001$.

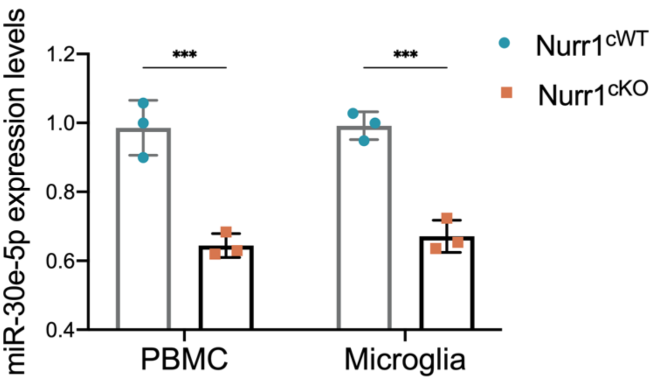

Supplementary Figure S3. Expression levels of miR-30e-5p in PBMC and microglia of Nurr1^{cKO} and Nurr1^{cWT}. n = 3 per group. Unpaired t test: ***p < 0.001.

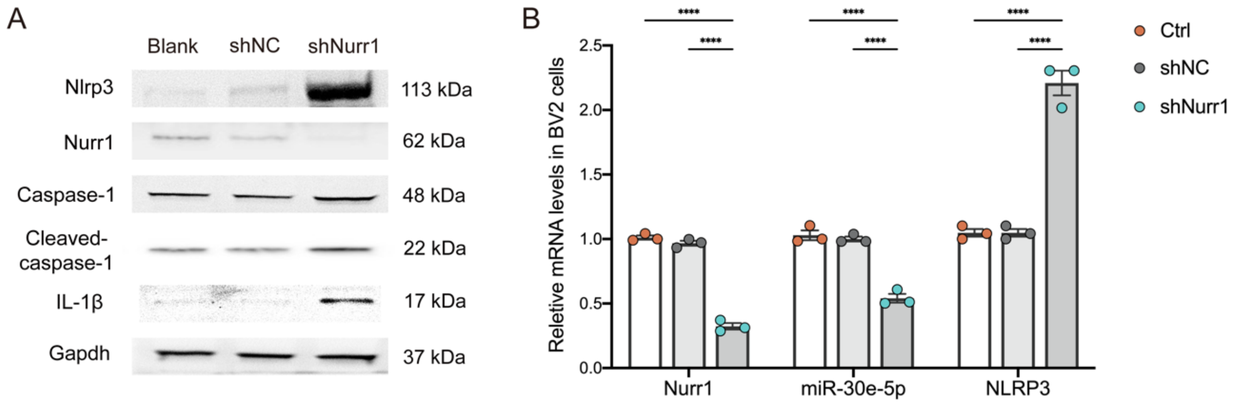

Supplementary Figure S4. Stable Nurr1 knockdown in BV2 microglia. Both Western blotting **(A)** and qRT-qPCR **(B)** results showed the successful knockdown of Nurr1 in the BV-2 cell line. One-way ANOVA: Tukey's multiple comparison test, ****p < 0.0001.
